# An Account of Diseases in an Eastern District of London, from November 20, to December 20, 1808

**Published:** 1809-01

**Authors:** 


*An Account of Diseases in an Eastern District of London, from November 20, to December 20,* 1808.

| | | | |
|---|---|---|---|
| ACUTE DISEASES. | | Dismenorrhœa | |
| Scarlatina - - - - - - | 3 | Amenorrhœa - - - - - | 5 |
| Peripneumonia - - - | 4 | Hysteria - - - - - - | 2 |
| Morbilli - - - - - - | 3 | Chorea - - - - - - - - | 1 |
| Erysipelas - - - - - - | 1 | Rheumatismus Chronicus | 12 |
| Rheumatismus Acutus | 2 | PUERPERAL DISEASES. | |
| CHRONIC DISEASES. | | Menorrhagia Lochialis | 3 |
| Tussis - - - | 22 | Dysuria - - - - - - | 2 |
| Tussis cum Dyspnœa | 19 | Ephemera - - - - - - | 4 |
| Pleurodyne - - - - | 4 | Abscessus Mammæ - - | 3 |
| Hydrothorax - - - - | 3 | Rhagas Papillæ - - - | 3 |
| Pyrosis - - - - - - | 1 | Diarrhœa - - - - - - | 5 |
| Vomitus - - - | 3 | INFANTILE DISEASES. | |
| Gastrodynia - - - - | 7 | Ophthalmia Purulenta | 3 |
| Dyspepsia - - - - - - | 8 | Tabes Mesenterica - - | 1 |
| Enterodynia - - - - | 5 | Herpes . - - - - - - | 2 |
| Diarrhœa - - - - - - | 4 | Diarrhœa - - | 7 |

The very sudden change in the temperature of the air which has taken place within a few days, and the extreme degree of cold that has prevailed, have produced very sudden effects in the vegetable world as well as in the human constitution. In a very few hours many plants, which were in perfect health, and screened in the greenhouse from the immediate effects of the external air, were almost destroyed. The effect on the human frame has also been considerable, and is likely to be followed by alarming, alarming, if not fatal consequences; particularly amongst persons far advanced in life, and who have for some time been liable to diseases of the chest at this season of the year.

The sudden change of weather has protracted the continuance, or occasioned the return of the different species of rheumatism, and particularly that affection of the muscles of the face described in a former report, has in several instances recurred.

